# The application framework of big data technology in the COVID-19 epidemic emergency management in local government—a case study of Hainan Province, China

**DOI:** 10.1186/s12889-021-12065-0

**Published:** 2021-11-04

**Authors:** Zijun Mao, Qi Zou, Hong Yao, Jingyi Wu

**Affiliations:** 1 College of Public Administration, Huazhong University of Science and Technology, No 1037 Luau Road, Hongshan District, Wuhan, 430074 Hubei China; 2 Non-traditional Security Institute, Huazhong University of Science and Technology, Wuhan, 430074 Hubei China

**Keywords:** COVID-19, Emergency management, Big data technology, Application framework, Local government, Hainan Province, China

## Abstract

**Background:**

As COVID-19 continues to spread globally, traditional emergency management measures are facing many practical limitations. The application of big data analysis technology provides an opportunity for local governments to conduct the COVID-19 epidemic emergency management more scientifically. The present study, based on emergency management lifecycle theory, includes a comprehensive analysis of the application framework of China’s SARS epidemic emergency management lacked the support of big data technology in 2003. In contrast, this study first proposes a more agile and efficient application framework, supported by big data technology, for the COVID-19 epidemic emergency management and then analyses the differences between the two frameworks.

**Methods:**

This study takes Hainan Province, China as its case study by using a file content analysis and semistructured interviews to systematically comprehend the strategy and mechanism of Hainan’s application of big data technology in its COVID-19 epidemic emergency management.

**Results:**

Hainan Province adopted big data technology during the four stages, i.e., migration, preparedness, response, and recovery, of its COVID-19 epidemic emergency management. Hainan Province developed advanced big data management mechanisms and technologies for practical epidemic emergency management, thereby verifying the feasibility and value of the big data technology application framework we propose.

**Conclusions:**

This study provides empirical evidence for certain aspects of the theory, mechanism, and technology for local governments in different countries and regions to apply, in a precise, agile, and evidence-based manner, big data technology in their formulations of comprehensive COVID-19 epidemic emergency management strategies.

## Background

Since the end of 2019, COVID-19, of an unknown origin, has spread rapidly in most countries and regions around the world. As of January 2021, the number of infected people is tens of millions, with 300,000 new cases every day [1]. The data show that COVID-19 continues to spread globally, and most countries and regions in the world are still in a state of public health emergency. The rapid outbreak of COVID-19 has posed a serious threat to the lives, health and safety of people worldwide.

A response to the COVID-19 epidemic has become the most important priority for governments of various countries and regions, testing the response and governance capabilities of governments. However, traditional emergency management measures are strained in the face of the COVID-19 epidemic, which is highly infectious, widespread, and complicated. Many scholars have noticed the necessity of applying big data technology to the COVID-19 epidemic emergency management and have highlighted the need to adopt a more agile, transparent and participatory approach in responses to the COVID-19 epidemic to compensate for the limitations of traditional epidemic emergency management [2, 3]. A strong empirical analysis and evidence-based practice supported by big data can effectively improve the scientificity of epidemic emergency management [3, 4]. In practice, many countries have actively adopted big data technology in all aspects of their responses to the COVID-19 epidemic.

However, because of the rapid emergence of the COVID-19 epidemic, the theoretical research and practical exploration of big data technology application have continued to focus mainly on the individual aspects of emergency management and thus lack a holistic, systematic thinking or design. Coping strategies are also fragmented and decentralized. Based on this situation and the perspective of emergency management, our research aims to comprehensively and systematically explore how big data technology can play a role in the entire process of the COVID-19 epidemic emergency management.

### Theoretical framework

With respect to the COVID-19 epidemic emergency management strategies in various countries and regions, problems of fragmentation and decentralization abound to varying degrees. For example, related practices focused on treatment over prevention, and the incomplete laws, imperfect emergency plans, slow early warning response speeds, insufficient interdepartmental coordination have made it difficult to curb the spread of COVID-19. Cannikin Law believes that the amount of water a wooden barrel can hold does not depend on the longest wooden board but on the shortest one. Similarly, in the practice of the COVID-19 epidemic emergency management, all the processes and elements of emergency management are crucial, and any aspect that is lacking often determines the effectiveness of an overall emergency management capability.

The theory of holistic governance suggests that public management should focus on the overall operation of a government’s internal agencies and advocates that management should be guided by public needs, which are located on various spectrums, from decentralization to concentration, from partial to overall and from fragmentation to integration [5, 6]. Hence, the process of the COVID-19 epidemic emergency management should be based on the perspective of holistic governance and should focus on improving the governance capabilities of all links and elements of an emergency management lifecycle and the capabilities there of by addressing shortcomings.

Emergency management is a cyclical dynamic process of multilink, multidepartmental collaboration and multiperson cooperation where different stages of emergency management produce different decision-making issues, and the activities implemented in each stage need to be carried out by strictly following the relevant emergency lifecycle [7]. Emergency management lifecycle theory provides a theoretical basis for determining the main stages of epidemic emergency management and their basic activities. The research on emergency management lifecycle theory has a long history, and many scholars have proposed different theoretical models, the most popular of which is the four-stage theory originally proposed by the National Federation of Governors of the United States [7, 8].

**Fig. 1:**
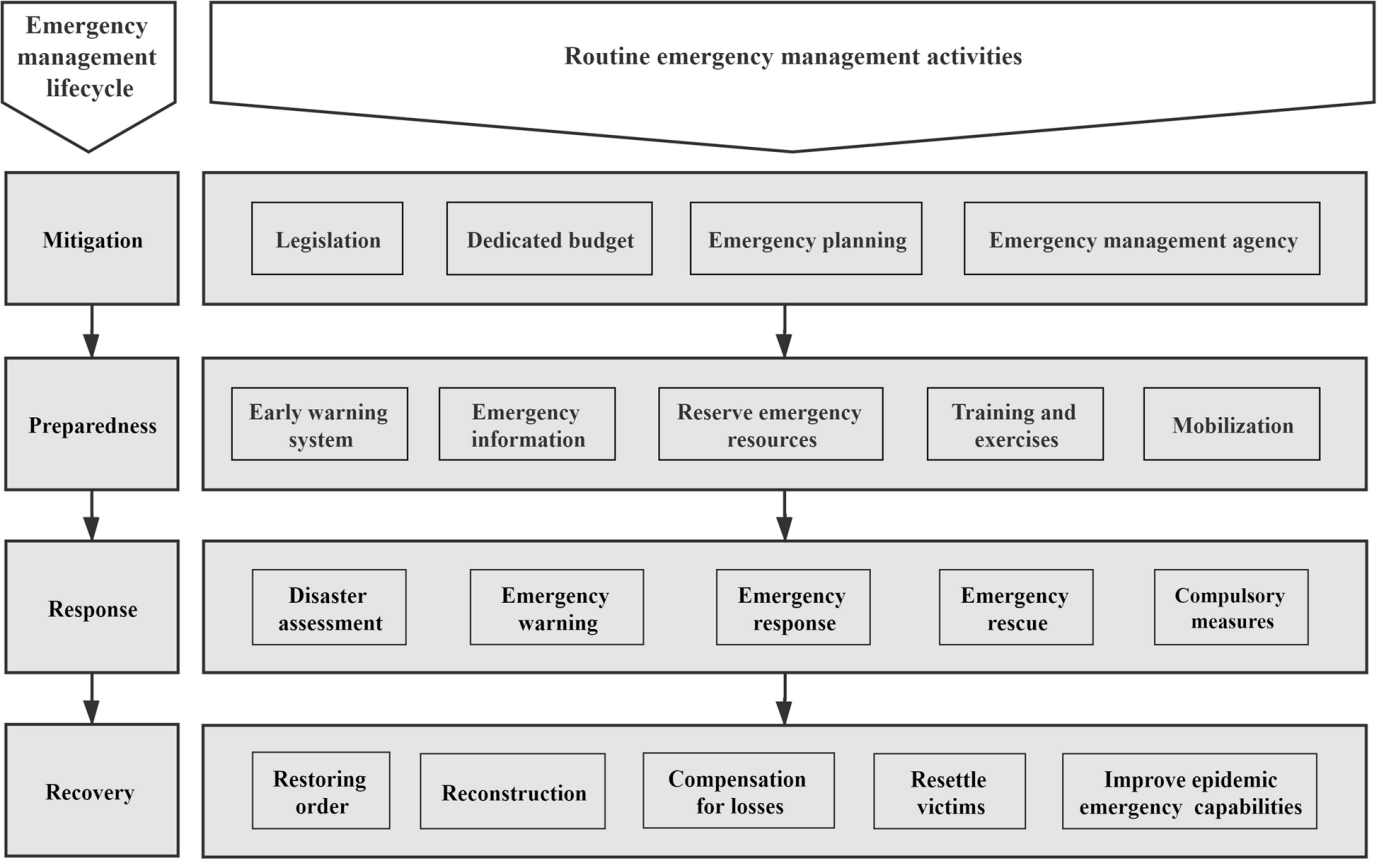
Emergency management lifecycle theoretical framework

Although many relevant studies have different definitions for the four stages of emergency management, they can be roughly summarized as shown in Fig. 1. These stages include 1) Mitigation, which is designed to eliminate or reduce the effects of hazards through legislation, budgets, formulations of emergency management plans and establishments of emergency management agencies; 2) Preparedness, which involves determining the extent of mitigation or disaster prevention measures and typically includes installing early warning systems, collecting emergency information, storing food, reserving medical and other emergency resources, emergency exercises, and mobilizing emergency personnel on standby; 3) Response, which involves response measures to be taken in the event of an emergency or disaster and includes initiating emergency warnings, implementing emergency responses, providing emergency aid to victims, adopting compulsory measures for specific areas or populations, assessing losses, etc.; and 4) Recovery, whereby areas affected by emergencies or disasters are assisted in rebuilding their physical infrastructures and in the restoration of their populations’ emotional and physical health through the reinstatement of normal activities, postdisaster reconstruction efforts, compensations for losses, resettlements and improvements in emergency response capabilities through education [7, 8].

The theoretical perspective of the emergency management lifecycle overcomes the limitations of fragmentation and decentralization in the practice of epidemic emergency management, providing a basis for all elements of epidemic prevention and control and the entire process. It provides us with an important theoretical foundation and framework for analysing and examining epidemic emergency management activities, which is conducive to the formation of a rapid response chain for public health emergencies and to the improvement of the pertinence and effectiveness of all epidemic emergency management measures.

For this study, emergency management lifecycle theory helps determine the basic activities of epidemic emergency management and the targets for the application of big data technology; that is, emergency management lifecycle theory helps us establish the application framework for an epidemic emergency management supported by big data technology.

### Application framework

Based on the theoretical framework, this chapter analyses the application framework of SARS epidemic emergency management, which lacked the support of big data technology. As a comparison, the application framework of the COVID-19 epidemic emergency management, supported by big data technology, is then addressed to clearly show the specific role and function of big data technology in COVID-19 epidemic emergency management.

### Application framework which lacked the support of big data technology: China’s 2003 SARS epidemic emergency management

**Fig. 2:**
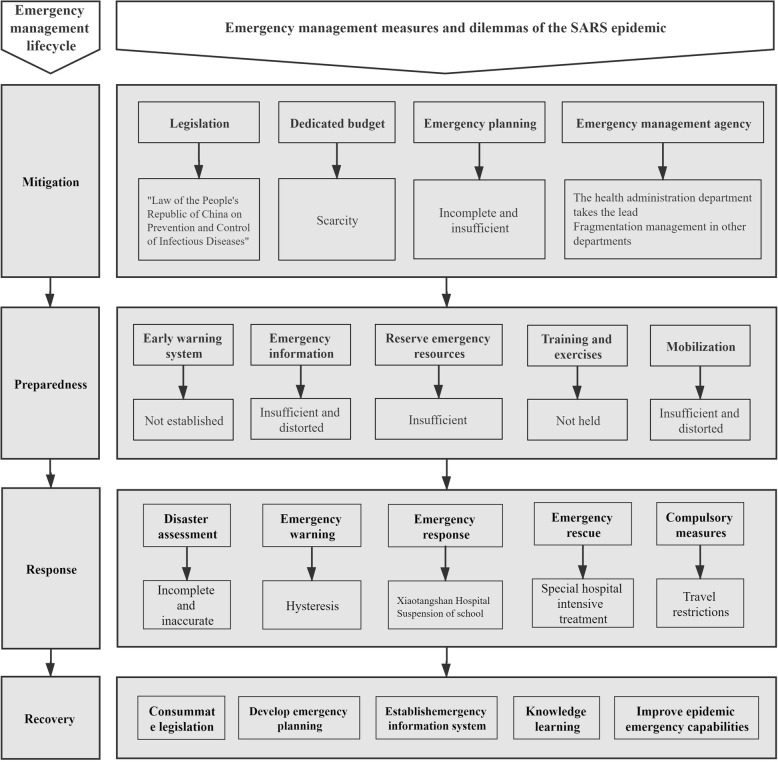
Application framework of China’s SARS epidemic emergency management which lacked the support of big data technology

At the end of 2002, SARS spread around the world, after which it was gradually eliminated and then eradicated in mid-2003. China’s SARS epidemic emergency management provided experience and lessons for the COVID-19 epidemic emergency management. This research applies emergency management lifecycle theory to analyse epidemic emergency management, summarize the application framework of the SARS epidemic emergency management, which lacked the support of big data technology, in China in 2003, to generalize the main contents and strategies of each stage of epidemic emergency management and to analyse the limitations of epidemic emergency management without the support of big data technology, as shown in Fig. 2. The detailed process of the four stages is described below.

In the mitigation stage of the SARS epidemic emergency management, China promulgated the “Law of the People’s Republic of China on the Prevention and Treatment of Infectious Diseases” in 1989 [9]. The State Council of China also issued the “Measures for the Implementation of the Law of the People’s Republic of China on the Prevention and Treatment of Infectious Diseases” in 1991 [10], which stipulated the laws and regulations that governments at all levels must carry out and abide by for the prevention, reporting, control, supervision and accountability of epidemic prevention and treatment and provided a legal basis for SARS epidemic emergency management. The law and regulations clarified that the health administrative department and its directly affiliated epidemic prevention agencies were responsible for the prevention and treatment of infectious diseases, while other government agencies were responsible for eliminating the threat of infectious diseases within their respective scopes, which provided an organizational and mechanistic basis for SARS epidemic emergency management [9, 10].

However, before the SARS epidemic outbreak, China had no authoritative agencies to address emergency incidents. Amidst the SARS epidemic, the health administrative department lacked the sufficient authority and capacity to lead multiple agencies to conduct the SARS epidemic emergency management. A specific example is that China’s epidemic information disclosure mechanism is imperfect. The Infectious Disease Prevention and Control Law stipulates that the power to publish major epidemic information is wielded by the State Council [9, 10]. Epidemic information often takes a long time to develop from internal government reporting and decision-making to its release to the public. The scarecity of a dedicated government budget and emergency plan, from the legislative level to the administrative level, is another constraint of the SARS epidemic emergency management.

In the preparedness stage of the SARS epidemic emergency management, when the first case of unknown pneumonia occurred in Guangdong Province in early 2003, Guangdong Province sent an expert team to investigate and issued investigation reports to all medical units in the province, requesting that the relevant units pay attention to the prevention and control the disease [11]. A month later, when the disease had spread on a small scale, China’s deputy minister of health led experts to Guangdong to help find the cause and guide the prevention and treatment of the disease [11].

However, local governments in China, especially those in the central and western regions with poor economic foundations, lacked the financial resources, materials and equipment needed for epidemic emergency management. There was also a lack of special epidemic response funds at the national and local levels, which to some extent restricted the smooth implementation of epidemic emergency management [12]. Before the SARS epidemic broke out, China had no epidemic information system, was unable to effectively collect and share epidemic information, and lacked a systematic emergency resources reserve, training, and exercises and mobilization. China’s lagging information disclosure mechanism made it difficult for the public to participate in emergency management in the preparedness stage of the SARS epidemic. The information suppression caused by the overconcentration of information disclosure power left local governments able to carry out only very limited patient treatment work in the early stages of the epidemic. The dual effects of insufficient information communication and information suppression largely contributed to chaos in the early stage of the SARS epidemic.

In the response stage of the SARS epidemic emergency management, after the epidemic attracted the attention of governments at all levels in China, they exerted their strong organizational capabilities to mobilize emergency resources for the treatment and isolation of confirmed patients [12]. In April 2003, the National Tourism Administration issued a notice restricting local tourism agencies from organizing citizens to travel to the central and western regions and rural areas to prevent the spread of the epidemic from these areas through tourism [11, 12]. Subsequently, Beijing, which was more severely affected by the epidemic, took measures to suspend classes in primary and secondary schools [11]. However, in the initial stage of the SARS epidemic, Chinese local governments failed to promptly collect effective data to assess the severity of the epidemic accurately, and some agencies even concealed information and failed to report it. The long chain of information reporting, decision-making, and disclosure resulting from information suppression aggravated this process. The imperfect information disclosure mechanism and early-warning mechanism led to the failure to stop the epidemic early [12].

In the recovery stage of the SARS epidemic emergency management, Chinese governments at all levels learned lessons through experiences, and the central government began to establish an effective nationwide epidemic emergency management system. The State Council promulgated the “Regulations on Public Health Emergencies”, which clarified the normative procedures for management of public health emergencies and the responsibilities, rights and obligations of various governments in emergency management. Furthermore, the regulations stipulated that governments at all levels should establish nationwide provincial emergency contingency plans and proposed the establishment of an epidemic-based emergency report system and an information reporting system. As one of the most affected areas, Guangdong learned lessons from the epidemic and set up a sensitive, efficient, unified and informationized epidemic emergency response mechanism, as well as an efficient information network and a complete provincial monitoring and control network [12].

In general, we find that through the joint efforts of its government, society and citizens, China finally overcame the SARS epidemic. Its experiences with this epidemic taught China lessons, and the government gradually established and improved the relevant laws, organizations, mechanisms and emergency plans for epidemic emergency management, laying a foundation for the success of China’s COVID-19 epidemic emergency management. However, due to China’s lack of preparation for epidemic emergency management through holistic lifecycle thinking and governance, all stages of its SARS epidemic emergency management exposed problems of varying degrees. In particular, the epidemic data collected by relevant agencies had problems. They were of insufficient quantity, single-sourced, of insufficient authenticity, and lacked sufficient real-time performance information; these data could not meet the requirements of complex network analyses involving people, public places, and regions, resulting in insufficient data value. Because the distribution of power over and responsibility for epidemic emergency between the central and local government was unclear, the corresponding decentralized and fragmented epidemic information communication systems and networks caused the underreporting and delayed reporting of epidemic information. Moreover, the epidemic information disclosure mechanism, with its excessively concentrated power, could not mobilize relevant regions and the public to fully participate in emergency management work in the early stage of the epidemic, which ultimately led to chaos in the emergency management system at the beginning of the SARS epidemic.

These limitations left local government unable to generate effective decision-making information and knowledge, and the epidemic information was suppressed within the central government and kept from the public, resulting in the lack of epidemic research and judgement and the slow response speed and inefficiency of China’s SARS epidemic emergency management. Without a rapid formation of an effective epidemic emergency management and command system, a misjudgement was caused by information distortion within the government and reduced the timeliness, accuracy, and authenticity of information disclosures between the government and the public. Therefore, the whole process of deployment—one based on emergency management lifecycle theory and the use of digital technology represented by big data technology in epidemic emergency management at different stages for accurate, agile, and systematic measures—is an important trend and a direction for improving overall epidemic emergency management capabilities.

### Application framework with the support of big data technology: COVID-19 epidemic emergency management

**Fig. 3:**
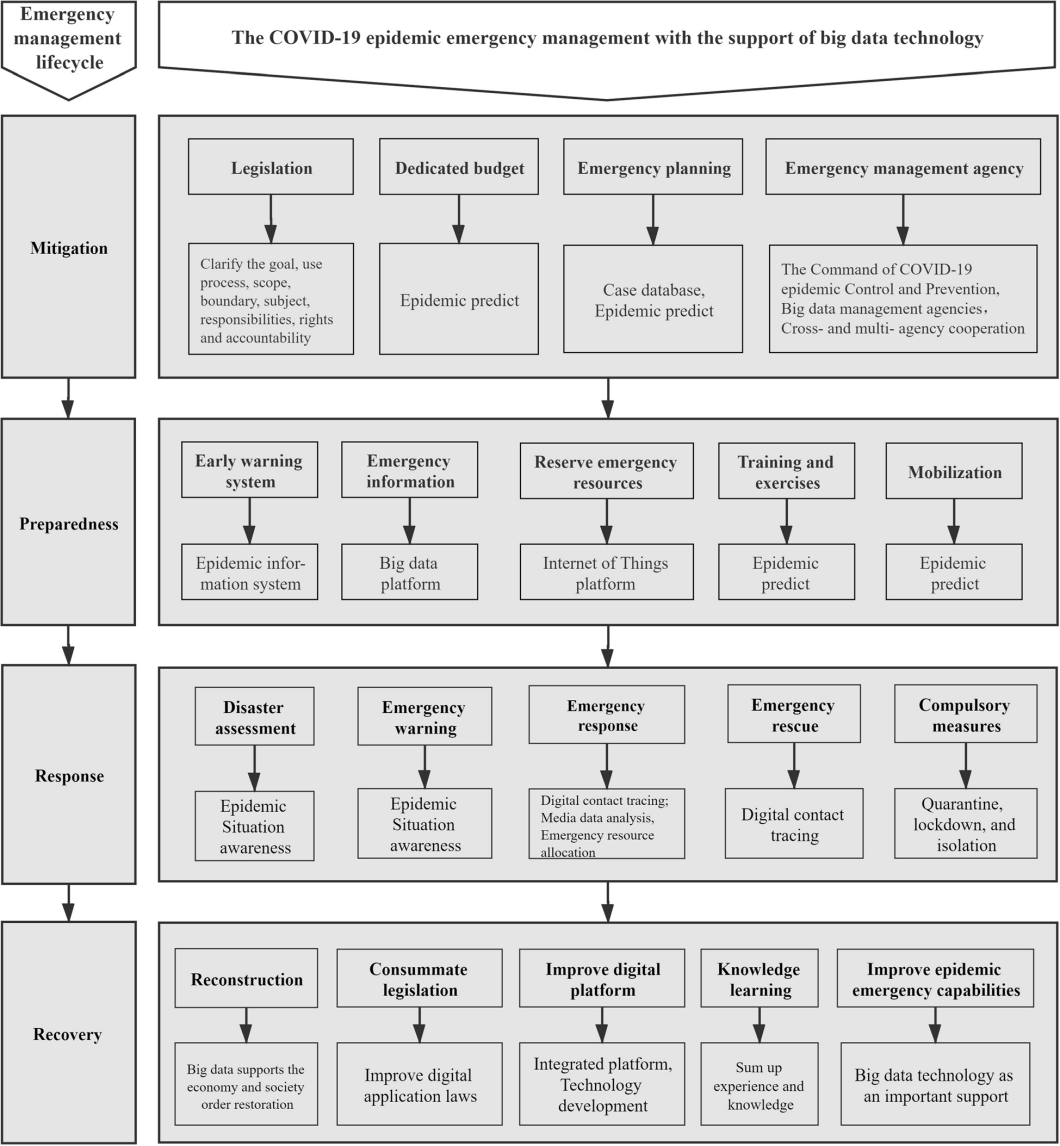
Application framework of the COVID-19 epidemic emergency management with the support of big data technology

Based on the theoretical framework and the application frameworks of epidemic emergency management, this research systematically reviews the relevant literature on the application of big data technology to the COVID-19 epidemic emergency management to summarize the experiences and lessons of China and other countries in this process. The literature search was conducted in October 2020 using the Web of Science and Chinese central and local governments’ websites. The search results were updated to include more recent literature as of July 2021. The keywords included terms to capture concepts associated with big data technology such as digital technology, artificial intelligence, the Internet of Things, emergency management, and COVID-19. Articles and government documents published between 2019 and the date of the search were included. Then, the technology, policies, management conditions and scopes of application of big data technology in the COVID-19 epidemic emergency management were reviewed. Finally, the entire and all-element process application framework of big data technology in the COVID-19 epidemic emergency management was proposed. The application framework is shown in Fig. 3.

Specifically, in the mitigation stage of the COVID-19 epidemic emergency management, legislation and government agencies should establish laws, regulations, systems, policies, organizational structures and operation mechanisms to apply big data technology. The legislative branch should formulate laws and regulations on the application of big data technology in epidemic emergency management to clarify the goals, use processes, scopes, boundaries, subjects, responsibilities, rights and accountabilities. Government agencies should establish or designate relevant agencies, by following the law, to enhance responsibility for the application of big data technology in epidemic emergency management and establish a clear cross- and multi-agency cooperation mechanism. In 2006, China promulgated the “Administrative Measures for the Monitoring and Reporting of Public Health Emergencies and Epidemics of Infectious Diseases”, which put forth the establishment of a public health information management platform, a basic health resource database and management application software to meet the needs of information collection, analysis and reporting for public health emergency management from the central to the local levels [13]. In China, the health administration is responsible for the supervision and guidance of the public health information management system, while medical institutions and disease prevention and control institutions at all levels are responsible for reporting [13]. Regarding the disclosure of epidemic information, China revised the Law on the Prevention and Control of Infectious Diseases in 2005 and 2013. These two versions unanimously delegated part of the right to disclose epidemic information to provincial governments, stipulating that each provincial government needs to regularly release information on common infectious diseases, including atypical pneumonia, to the public, but the power of information disclosure for new major infectious diseases remained with the State Council [9, 14].

Establishing a unified and efficient epidemic command agency is another focus of the COVID-19 epidemic mitigation stage. Thus, the Command of COVID-19 epidemic Control and Prevention, an official group set up by Chinese governments at all levels, led and coordinated relevant agencies to utilize big data technology for the COVID-19 epidemic emergency management. Under the guidance of the Command of COVID-19 epidemic Control and Prevention, the big data management agencies in Zhejiang Province, Guangdong Province, Hainan Province and other places in China provided support for big data technology application in the COVID-19 epidemic emergency management.

These activities provided a legal and organizational basis for the application of big data technology in the COVID-19 epidemic emergency management. In addition, authorities can apply big data technology to predict the local outbreak probability, time and intensity of the COVID-19 epidemic, simulate the spreading process, and help to accurately prepare the emergency management budget for the epidemic [15]. Big data technology can also be applied to build a rich and detailed case database of epidemic emergency management, which facilitates a rapid formulation of COVID-19 epidemic emergency plans.

In the preparedness stage of the COVID-19 epidemic emergency management, government agencies and social organizations should gather multiple forces to build a big data platform, an Internet of Things platform and a public health information system to provide a digital foundation for the management of an early warning system, epidemic information, and emergency. The use of big data technology to improve the prediction and early warning capabilities of a public health surveillance system is essential for epidemic emergency management. In addition, a digital and intelligent management of emergency resources (such as disease control experts, medical staff, medical resources, and food) based on Internet of Things technology also has a positive effect on improving the rapid response of epidemic emergency management.

After the SARS epidemic ended in 2004, China established a cross-level epidemic information system and a GIS-based public health information system to coordinate information collection, data analysis and decision-making among multiple agencies [13, 16]. The Korean Centre for Disease Control and Prevention built a COVID-19 Smart Management System based on big data, such as security camera footage, credit card records and even the GPS data of cars and mobile phones, that can be used to track people who may be exposed to COVID-19 and impose quarantine measures on them [17]. Due to its interoperability and shareability, big data technology in the COVID-19 epidemic emergency management can foster a national and provincial epidemic surveillance system, which help build an integrated coordination mechanism connecting the central and local governments and all government departments to improve administrative preparedness for epidemic emergency management [18, 19]. In contrast to a mobile application-based digital contact tracing of users’ autonomous data, a digital COVID-19 epidemic platform based on big data technology gathers and analyses real-world data, such as medical care, transportation, communication, consumption, positioning, and social media information, to reduce or even eliminate digital discrimination against marginalized groups while enhancing citizen participation and providing sufficient political preparedness for emergency management [3, 18]. Instead, the important agenda is to improve the legal framework, the governance framework and social compliance, while fully protecting the privacy of the public and ensuring timely disclosures of epidemic information from the legal, organizational and technical levels [18]. Moreover, big data technology helps local governments predict the outbreak probability, spread speed and scale of the COVID-19 epidemic to create more precise mobilization decisions regarding emergency exercises, mobilizations of emergency forces and the management of emergency resources [15, 20–23].

However, in China, information suppression still existed in the preparedness stage of its COVID-19 epidemic emergency management. When sporadic pneumonia of an unknown cause occurred in Wuhan in December 2019, according to the epidemic information disclosure mechanism of the Infectious Disease Prevention and Control Law, the municipal and provincial governments lacked the power to disclose this new epidemic to the public. The release of epidemic information by a local government to the public needed to follow the chain of “report to the central government decision-makers, and then central government authorizes local government disclosure”. Furthermore, the authorities were not clear about the cause and infectious ability of this unknown pneumonia, which led to the lack of timely and effective emergency management measures and the rapid spread of COVID-19 across the country.

In the response stage of the COVID-19 epidemic emergency management, big data technology is used for epidemic situational awareness. This facilitates damage assessment, emergency warning, dynamic monitoring, emergency rescue and the implement of compulsory measures. Mainland China developed a smartphone applet to collect residents’ self-reported health status, travel history and other information, used big data analysis and algorithm technology to track infected populations and their close contacts, and calculated personal risk assessment results to generate health QR codes for personal risk identification [17]. Hong Kong and Taiwan’s containment strategies focused on isolating imported cases [3]. Hong Kong designed a compulsory electronic wristband and a mobile application for imported passengers [17]. All passengers entering Hong Kong were required to wear it. The device alerted the authorities whenever passengers left their designated isolation place [17]. Taiwanese authorities used the National Health Insurance database and the Immigration and Customs database to match symptoms with travel experiences to identify people who may have been exposed to COVID-19 [17]. Although Western countries, such as Europe and the United States, did not adopt big data technology to track confirmed cases at the national level, Google, Apple, MIT and some European companies designed apps based on Bluetooth technology that can inform users when they contact confirmed patients [3, 24]. Adopting big data technology to analyse social media to understand public sentiment and opinion in epidemic emergencies helps to improve direct communication between the government and the public, maximize public participation and public compliance, and improve the government’s response to public needs in an emergency [25, 26]. Furthermore, big data technology, such as the Internet of Things, helps accurately allocate emergency resources to further improve responses to the COVID-19 epidemic [23]. Finally, a situational awareness of the COVID-19 epidemic based on big data technology, combined with active traditional emergency management measures such as contact tracing, quarantine, treatment, lockdown and isolation, greatly improves the responsiveness and accuracy of epidemic emergency management [27, 28]. In other words, a digital emergency response system that connects national government levels, government agencies, and governments in general to society plays a vital role in epidemic warning, monitoring, and evidence-based responsive decision-making [18, 26].

In the recovery stage of the COVID-19 epidemic emergency management, big data technology is applied to support post-epidemic reconstruction, work resumption and the reopening of schools, thereby improving a government’s emergency capacity, urban resilience and rapid response capacity for public health emergencies [29, 30]. For example, during the post-COVID-19 epidemic period in China, the health QR codes based on big data technology were commonly used as certificates for citizens to travel, return to work and return to school [24]. Big data technology played an important role in distributing economic assistance to impoverished patients and consumer subsidies to citizens in the post-epidemic period in China. Finally, at this stage, government departments should summarize their experience and knowledge and accelerate the promotion of legislation, construction of platforms, and development of technology to apply big data in epidemic emergency management.

Reforming the information disclosure mechanism was another crucial measure taken by China in the recovery stage of its COVID-19 epidemic emergency management. The National Health Commission issued the “Law of the People’s Republic of China on the Prevention and Control of Infectious Diseases (Revised Draft for Solicitation of Comments)” in October 2020 to solicit opinions from society and the public. The draft clarified that the application of big data technology supported the emergency management of the epidemic and clearly pointed out that “when an infectious disease breaks out or spreads, local governments at or above the county level should promptly and accurately disclose local epidemic information including but not limited to the name of the infectious disease, the spread of the epidemic, and the number of confirmed, suspected, and dead cases of infectious diseases” [31]. The draft devolved the power to disclose epidemic information from the State Council to local governments at the county level and above and made it clear that even if the published epidemic information was later excluded, the responsible officials would not be held accountable.

This major change played a significant role in the delta variant’s spread in Guangzhou, Hangzhou, Wuhan, Xiamen and other places in China from May to September 2021. These cities, facing the discovery of new cases of the delta variant, provided early warnings to the public within 24 h without needing the approval of the central and provincial governments. They also disclosed the trajectory information of all confirmed cases and their contacts, collected by digital contact tracing, within 48 h [32]. These practices helped China restrict the spread of the delta variant to a very limited area and to quickly eliminate the second wave of the COVID-19 epidemic.

By comparing the SARS epidemic emergency management which lacked the support of big data technology and the COVID-19 epidemic emergency management with the support of big data technology, we find that big data technology, with its velocity, volume and variety, enables government agencies and medical institutions to extract useful information from huge amounts of epidemic data to provide support for emergency management decisions. Big data technology empowers epidemic emergency management, which is of great significance in enhancing a government’s and society’s epidemic emergency capacity and efficiency.

### Case verification of the application framework

Our research conducted a case study on the use of big data technology for the COVID-19 epidemic emergency management in Hainan Province, China, to verify the feasibility, effectiveness and value of the application framework we proposed and to analyse its application prospects and possible practical obstacles. We collected and analysed policies, regulations and documents to fully understand the entire process of the COVID-19 epidemic emergency management in Hainan Province. At the same time, we investigated and interviewed the COVID-19 epidemic emergency management participants to systematically comprehend the strategy and mechanism of applying big data technology in each of the four stages of the COVID-19 epidemic emergency management in Hainan Province.

## Methods

### Case selection and study

Hainan Province is the southernmost provincial administrative region in China, with Hainan Island as its main body. The total land area of Hainan Province is 35,400 km^2^, of which the mainland area of Hainan Island is 33,900 km^2^. Hainan Island is separated from the Leizhou Peninsula in Guangdong Province by the Qiongzhou Strait, which is approximately 18 nautical miles wide [33]. With its tropical island scenery, Hainan Province become one of the most popular tourist destinations for Chinese and global tourists, and in 2019, the province attracted 83.112 million tourists [34]. However, the permanent population of Hainan Province at the end of 2019 was only 9.45 million [31]. The nature of its tourist attractions has led Hainan Province to have a very high proportion of floating populations and a rather high flow rate.

Therefore, the geographical location of its island makes the importation of COVID-19 cases a priority and highlights the difficulty of the COVID-19 epidemic emergency management. However, Hainan Province, with its strong COVID-19 epidemic emergency management capabilities, has outstandingly curbed the spread of COVID-19 and has become one of the provinces least affected by the COVID-19 epidemic in China.

Specifically, as of August 7, 2020, the province had a total of 171 confirmed cases of COVID-19, including 165 discharged cases and 6 deaths. The vast majority of cases were concentrated between January 22 and February 19, 2020 (168 cases), and only 3 cases appeared in the following half-year [3, 35]. These figures prove the success of the emergency management strategy for the epidemic in Hainan Province, even though Hainan Province, as a popular tourist destination for tourists, faces a greater and uncertain threat from COVID-19. Hainan Province is one of the first provinces in China to use big data technology for its COVID-19 epidemic emergency management [29]. Its successful experience became the basis for our research and our case selection.

### File collection and analysis

We sorted through the legal and institutional documents on public health emergencies, infectious disease management, generalized emergency management, government information management and big data management implemented at the national level in China and in Hainan Province. Based on these files, we tried to obtain a comprehensive understanding of China and Hainan Province’s specifications in applying big data technology for the COVID-19 epidemic emergency management in terms of the boundaries, processes, mechanisms, rights and obligations of subjects and objects and accountability systems. We also reviewed the public announcements, reports, policies, government documents, project planning documents and other documents of the Hainan provincial government relevant to this research topic, which included notifications, meeting resolutions and official document exchanges between the Big Data Administration and the Command of COVID-19 epidemic Control and Prevention, Public Security Administration, health administration, Centre for Disease Control and Prevention (CDC), railway management department, communication management department and other government agencies involved in the project, to help us further describe and fully understand the specific details of the application of big data technology for the COVID-19 epidemic emergency management in Hainan Province.

### Semistructured interviews

Based on the application framework we proposed, we conducted semistructured interviews with government staff and big data technicians of the big data application project for the COVID-19 epidemic emergency management in Hainan Province. First, we interviewed two project leaders to understand the plans, goals and implementation details of the big data technology project. Second, we interviewed three big data technicians to understand the entire process of the epidemic’s data planning, collection, storage, analysis, application and their technical details to understand big data and the background, content and application scenarios of technology development. Finally, we interviewed five government staff. On the one hand, through interviews with the staff of the Big Data Administration, we clarified the government’s work process and the cooperation and coordination mechanism of multiple agencies. On the other hand, based on the professions engaged with the COVID-19 epidemic, we also interviewed the staff of the Hainan Provincial Health Commission and CDC to understand the process and the application of big data technology for the COVID-19 epidemic emergency management. As a result, we conducted semistructured interviews with 10 participants in emergency management.

Before each interview, we ensured that interviewees fully understood the purpose of the interview, namely, Hainan Province’s workflow and the advantages and limitations of using big data technology for the COVID-19 epidemic emergency management. We recorded the interviews and converted the audio data into text data. Then, the main points in the interview materials were classified to summarize the application of big data technology for the COVID-19 epidemic emergency management in Hainan Province.

## Results

**Fig. 4:**
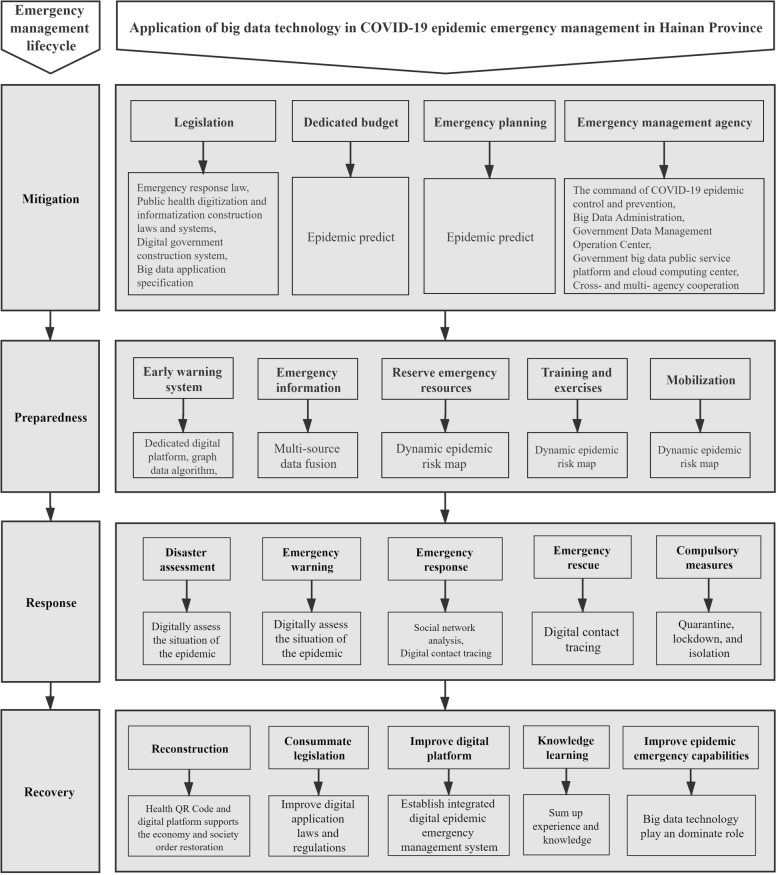
Application of big data technology in COVID-19 epidemic emergency management in Hainan Province

Based on the files and interview materials, we analysed the application of big data technology in the four stages of the COVID-19 epidemic emergency management: mitigation, preparedness, response and recovery. Figure 4 shows the application framework applied in Hainan Province for the COVID-19 epidemic emergency management. The details of the process are described below.

### Application of big data technology in the mitigation stage of the COVID-19 epidemic emergency management in Hainan Province

The mitigation stage emphasizes the measures taken before an epidemic emergency occurs. Before the COVID-19 epidemic, the government of Hainan Province attached importance to the application of big data technology for epidemics and issued a series of laws and regulations such as the “Regulations on Emergency Response to Public Health Emergencies” in 2006, “The ‘Smart Information Island’ Planning of Hainan Province (2010–2020)”, “The Ordinance on the Development and Application of Big Data in Hainan Province” in 2016 and “The Big Data of Hainan Province Regulations On the Development and Application” in 2019. In the field of epidemic emergency management, such documents demonstrate that government agencies should promote the application of big data to optimize the allocation of emergency resources and improve social governance and emergency management capacity for epidemic emergency management. Accordingly, the Big Data Administration and Government Data Management and Operation Center were established in 2019 [36].

After COVID-19’s first outbreak in Wuhan City, Hainan Province immediately established its Command of COVID-19 epidemic Control and Prevention as the highest leading authority for the COVID-19 epidemic emergency management. The Command required the Big Data Administration to integrate the superiority of big data technology into its COVID-19 epidemic emergency management. In other words, the Hainan Provincial Command of COVID-19 epidemic Control and Prevention played a leading and coordinating role, while the Hainan Big Data Administration played a leading as well as implementing role and other government agencies played a coordinating role, thereby forming an integrated organizational structure and coordination mechanism for the application of big data to manage the COVID-19 epidemic. Therefore, Hainan Province established the legal and organizational bases for applying big data technology to the COVID-19 epidemic emergency management. Furthermore, the Hainan provincial authorities used national COVID-19 big data, especially the epidemic information from Hubei Province, the earliest centre for infection, to analyse and predict the local spread of COVID-19, which was also the basis for formulating emergency budgets and emergency plans.

### Application of big data technology in the preparedness stage of the COVID-19 epidemic emergency management in Hainan Province

The preparedness stage emphasizes the preparatory measures taken by the government and society to anticipate the imminent occurrence of an emergency. The big data technology project of the COVID-19 epidemic emergency management in Hainan Province was initiated by the Command of COVID-19 epidemic Control and Prevention on January 29, 2020. The governor of Hainan Province instructed relevant government agencies to cooperate with the Big Data Administration to apply big data technology for the COVID-19 epidemic emergency management during the regular meeting of the Command of COVID-19 epidemic Control and Prevention. Earlier, the Hainan Provincial Big Data Administration had established the Provincial Government Big Data Public Service Platform and cloud computing centre. Based on this platform, the Hainan Provincial Big Data Administration immediately organized disease prevention and control experts and big data technicians to evaluate the emergency situation, policies and strategies, negotiated with the Command of COVID-19 epidemic Control and Prevention and other relevant government agencies to formulate the multisource big data to be used in determining strategic goals and specific implementation plans for high-risk populations, locations and areas, and submitted achievable data demands to the Command of COVID-19 epidemic Control and Prevention. The Command of COVID-19 epidemic Control and Prevention coordinated with relevant agencies to provide the designated data. The platform also allowed Hainan Province to construct a dynamic epidemic risk map based on the distribution of people from high-risk areas in various regions. An epidemic risk map provided evidence for the authorities to deploy emergency resources and mobilize emergency forces before the widespread outbreak of COVID-19. In addition, Hainan Province issued early warnings to the public based on the situation in Wuhan before the emergence of a confirmed patient and proactively released COVID-19 information so that Hainan Province could fully prepare for the epidemic.

The Hainan Provincial Big Data Administration collected the first batch of data, as shown in Table 1, from the Hainan Provincial Command of COVID-19 epidemic Control and Prevention, Public Security Administration, Health Commission and communications operators before February 10, 2020, and aggregated them into big data for an emergency response to the COVID-19 epidemic [3]. Big data storage requires a cloud storage platform with sufficient capacity. Under the guidance of the project leader, the technicians of the Hainan Provincial Big Data Administration integrated and cleaned up the data and then uploaded all databases to the Hainan Provincial Government Big Data Public Service Platform. The platform provides hive data warehouse tools to manage, extract, query and analyse big data [3].

**Table 1:** List of big data collection for the COVID-19 epidemic emergency management in Hainan Province

Database	Data provider	Description	Collection method
Resident tracking information database	Health Committee	Used to understand where citizens have gone and who they have contacted.	The Health Committee organizes the CDC, community health service centres, and community (village) residents’ committees to conduct household surveys and telephone verifications.
Confirmed cases information	Public Security Administration, Health Committee	Used to understand who was infected by COVID-19.	Reported by the CDC and other medical institutions.
Mobile phone signalling	Communications operator	Used to understand where people ultimately arrived.	Provided by China Mobile, China Unicom, China Telecom, and China Broadcasting Network Corp Ltd.
Information on high-risk groups and close contacts	Command of COVID-19 Control and Prevention	Used to understand their close contacts in confined spaces.	Summary of the CDC information.
Hospital fever information database	Health Committee	The relationship between fever patients and COVID-19 and their tracking.	The medical institutions report through the National Health Direct Reporting System.

Source: Mao Z, Yao H, Zou Q, Zhang W, Dong Y: Digital Contact Tracing Based on a Graph Database Algorithm for Emergency Management During the COVID-19 Epidemic: Case Study. JMIR MHEALTH UHEALTH 2021, 9(1):e26836. 10.2196/26836

### Application of big data technology in the response stage of the COVID-19 epidemic emergency management in Hainan Province

In the response stage of the COVID-19 epidemic emergency management, the graph database association graph analysis was applied to track key populations, the source of infection, and the transmission path of the epidemic, sequentially determine the infection risk and threat precisely. These analysis results were used by the authorities for the COVID-19 epidemic damage assessment, emergency warning and treatment. Specifically, the data analysis technicians of the Hainan Provincial Big Data Administration extracted data from the Hainan Provincial Government Big Data Public Service Platform and used the graph database Neo4j algorithm and ECharts data visualization tools to analyse the relationships network of people-people, people-transportation and people-public locations and areas to identify close contacts, high-risk locations and areas that were threatened by the COVID-19 epidemic. Finally, big data technicians adopted the ECharts data visualization tool to perform visual analysis and design an easy-to-use interactive operating system for non-technical personnel to query big data analysis results and obtain useful information for decision-making. Thus, the application of big data technology in Hainan Province can obtain information and knowledge from multisource data for different decision-makers, improve the response speed of the COVID-19 epidemic emergency management.

Based on the results of the first batch of big data analysis, the Hainan Provincial Big Data Administration was able to investigate the potential risk sources of COVID-19 epidemic, and successfully identified a group of close contacts, high-risk locations and areas. Among them, a new coronavirus patient was found who wasn’t found by traditional measures.

The Hainan Provincial Big Data Administration regularly updated data in batches and wrote COVID-19 epidemic prevention and control reports based on the results of big data analysis, and reported them to the Command of COVID-19 epidemic Control and Prevention. Under the overall leadership of the Command, the Health Administration, Public Security Administration, CDC, medical institutions, and grassroots residents’ committees were organized to track key populations, release early warning information to government agencies and the public in a timely manner, and strengthen cross-agency information sharing to carry out emergency management and adopt quarantine measures for key populations, personnel flow restriction and closure of high-risk places.

### Application of big data technology in the recovery stage of the COVID-19 epidemic emergency management in Hainan Province

When the COVID-19 epidemic emergency management entered the recovery stage, Hainan Province continued to fully apply big data technology to daily epidemic prevention and control. Compared with traditional epidemic information systems and data processing tools, an analysis via the graph database can quickly match complex network relationships, greatly improving computing efficiency. Big data platforms and visualization tools can quickly identify and process multisource big data to determine spatial and social network relations. Once a confirmed or suspected case appears, the Hainan Provincial Government can rely on the application of big data technology to update information and respond quickly. It can help different agencies formulate and apply scientific and effective measures to improve the rapid response capability of urban public health emergencies and resilience.

The Hainan Provincial Government Big Data Public Service Platform and the application of the graph database analysis method can continuously update epidemic data through dynamic real-time monitoring. In addition, combined with the current use of the Hainan Provincial Health QR Codes for the entire population, the population can be tracked continuously, providing information support for the normal operations of a city and its government’s handling of the potential risks of COVID-19. Furthermore, the visual interactive operating system, based on big data technology, can show the correlation and interaction with people intuitively and dynamically. Because of its ease of operation, it is possible for the platform to achieve popularization at the decision-making and grassroots levels and to play an important role in epidemic prevention and control. Hainan Province strengthened the development and application of the health QR codes and an integrated digital epidemic emergency management system and further used big data technology to provide important support for the restoration of social and economic orders and postdisaster reconstructions.

Finally, Hainan Province is constantly updating and applying big data technology to normalize the management of the COVID-19 epidemic and improve relevant laws, regulations, organizational structures, mechanisms and platforms to improve governments’ big data processing and analysis capability to enhance epidemic emergency management capabilities.

## Discussion

### Main findings

Our research proposed a theoretical framework and application framework of big data technology that supports the four stages of the COVID-19 epidemic emergency management: migration, preparedness, response and recovery. Then, taking Hainan Province, China, as a case study, our research summarized the framework and mechanism of the COVID-19 epidemic emergency management supported by big data technology and verified the feasibility and value of the proposed application framework. By comparing the similarities and differences between the two frameworks, our research identified four trends in big data technology supporting the COVID-19 epidemic emergency management: 1) the mitigation stage, when a legal system is continuously improved, the system and mechanism move from decentralization to integration and concentration, from government-led to multi-participant collaboration, and an integrated digital platform is the driving force; 2) the preparedness stage, when data sources are changing from single to multiple, collection and analysis methods are changing from manual to intelligent, and emergency preparedness measures are changing from disordered to ordered; 3) the response stage, when monitoring, early warning and emergency responses shift from one-sided to comprehensive, from static to dynamic, and from extensive to precise; 4) the recovery stage, when, oriented by public demand, big data technology and products are applied to restore economic and social order, develop and improve an integrated digital epidemic emergency management system, and transition from comprehensive emergency responses in wartime to precise preventions in peacetime.

At the same time, the case study of Hainan Province has enriched the practical connotation of the application framework, expanded its application prospects, and highlighted areas for its improvement. The successful practical applications are shown in Table 2.

**Table 2:** Overview of big data technology application in the COVID-19 epidemic emergency management

Emergency management lifecycle	Emergency activities	Big data technology application measures
Mitigation	Legislation	1. Establish and improve laws and systems related to the digital transformation of epidemic emergency management. 2. Incorporate the digital transformation of epidemic emergency management into the legislation of digital government construction. 3. Clarify the goals, use processes, scopes, boundaries, subjects, responsibilities, rights and accountabilities of big data technology application in epidemic emergency management.
	Dedicated budget	Use big data technology-based epidemic risk prediction, situational awareness, and risk maps to formulate special dedicated budgets.
	Emergency planning	Use big data technology to establish an epidemic emergency management case database, and combine the results of epidemic risk prediction to formulate emergency plans.
	Emergency management agency	1. Establish a unified, supremely authorized epidemic prevention and control command to coordinate the application of big data technology in emergency management. 2. Establish a big data management administrative agency directly responsible for the digital construction of epidemic emergency management. 3. Establish a cross- and multi-agency collaborative governance mechanism. 4. Establish an emergency management mechanism involving government, society and the public, especially a communication mechanism.
Preparedness	Early warning system	1. Establish a dedicated, integrated epidemic emergency management digital platform. 2. Develop an epidemic prediction and warning system based on the digital epidemic emergency platform.
	Emergency information	1. Realize the real-time sharing of epidemic data based on port connections on the epidemic digital emergency platform. 2. Integration of public-private sector multisource epidemic big data, such as medical care, transportation, communication, consumption, positioning, and social media. 3. Use big data mining, storage, sharing and analysis technologies to process epidemic information, and realize real-time epidemic monitoring and information disclosure. 4. A local government can disclose the epidemic information to the public in a timely manner without obtaining authorization from a higher-level government.
	Reserve emergency resources	Use big data technology to support epidemic risk prediction and the Internet of Things platform to reserve emergency resources.
	Training and exercises	Use big data technology-based epidemic risk prediction to carry out emergency training and exercises.
	Mobilization	Use big data technology-based epidemic risk prediction to carry out emergency mobilization.
Response	Disaster assessment	Use the dynamics of epidemic situational awareness based on big data technology to assess the damage of the epidemic.
	Emergency warning	Use the dynamic epidemic situation awareness based on big data technology and the early warning system to issue emergency warnings to the public in a timely manner.
	Emergency response	1. Use digital contact tracing to find the source of the epidemic and take quarantine measures. 2. Use the internet of things platform to efficiently, quickly and accurately configure emergency resources. 3. Use big data technology to analyse traditional media and new media data to improve responsiveness to public opinion and public need.
	Emergency rescue	Use digital contact tracing to accurately identify patients and contacts, and conduct centralized treatment or medical observation on them.
	Compulsory measures	Use big data technology to cooperate with traditional emergency management measures such as contact tracing, quarantine, treatment, lockdown and isolation.
Recovery	Reconstruction	1. Use big data technology to support the economy and society order restoration. 2. Use big data technology to help severely affected groups and socially disadvantaged groups through precise economic assistance and subsidies.
	Consummate legislation	Strengthen the legislation for big data technology application in epidemic emergency management, especially to ensure public privacy, public rights and social compliance in the process of technology application, and improve transparency and accountability.
	Improve digital platform	Establish and continuously optimize the integrated digital epidemic emergency management platform.
	Knowledge learning	1. Summarize the successful experience and knowledge of big data technology application in the process of epidemic emergency management, and promote it at the national level. 2. Use big data technology to improve the case database of epidemic emergency management.
	Improve epidemic emergency capabilities	Take big data technology as an important element to improve the responsiveness of the epidemic emergency management system.

1. In the mitigation stage of the COVID-19 epidemic emergency management, Hainan Province enacted a series of laws and regulations that legally clarified the scope and specifications of big data technology to support epidemic emergency management. The laws and regulations that must be followed for big data technology development and application and relevant activities, such as big data development and sharing, big data application, data security protection and legal liability, were clarified by Hainan Province. Furthermore, the establishment of the Command of COVID-19 epidemic Control and Prevention, the Big Data Administration and the epidemic emergency management digital platform provide an organizational and platform basis for the application of big data technology to the COVID-19 epidemic emergency management. The application of big data technology in risk prediction can provide support for accurately formulating epidemic emergency budgets and emergency plans.

2. In the preparedness stage of the COVID-19 epidemic emergency management, the Command of COVID-19 epidemic Control and Prevention established by Hainan Province was headed by the governor. Because of its high administrative level and power, a series of epidemic emergency management measures could be enforced in the state of emergency response to the epidemic, and all government agencies as member units were required to cooperate. All government agencies could also communicate in a timely manner, thereby enabling the effective and synergistic implementation of epidemic emergency management. Based on this mechanism, the Hainan Provincial Command of COVID-19 epidemic Control and Prevention was able to promote the application of big data technology in the COVID-19 epidemic emergency management and take advantage of its authorization, its administrative orders and other metasynthetic management methods to form a collaborative and integrated rapid response mechanism. Subsequently, the Big Data Administration organized experts and technicians from relevant fields to assess the project, determine the specific implementation plan and data requirements, and complete the data collection, cleaning, aggregation, and storage required by the government’s big data public service platform, thereby enabling the full preparedness of the province for the project. The dynamic epidemic risk map drawn by big data technology provided an opportunity for authorities to distribute emergency supplies and mobilize emergency forces before the outbreak of COVID-19.

3. In the response stage of the COVID-19 epidemic emergency management, under the leadership of the Hainan Provincial Command of COVID-19 epidemic Control and Prevention, the big data technicians under the Big Data Administration adopted a graph database algorithm and an ECharts data visualization tool that used big data analysis technology to track hidden infected populations, suspected infected populations and their close contacts, to identify high-risk areas and locations, and to create a visualization window that could be operated interactively by a decision-maker, thereby providing evidence-based support for the rapid response to and scientific decision-making for the COVID-19 epidemic. Utilizing an integrated digital platform for epidemic emergency management to integrate and analyse big data from multiple sources is well-suited to emergency management measures such as contact tracing, quarantine, treatment, lockdown and isolation.

4. In the recovery stage of the COVID-19 epidemic emergency management, the application of big data technology allowed for the quick and agile tracking of key populations that could appear at any time to apply epidemic emergency management measures to specific populations and places accurately, without affecting the normal economic order and people’s livelihoods. The interactive operating system, based on data visualization, made it easier for decision-makers with different professional backgrounds and relevant grassroots staff to use big data technology to formulate epidemic emergency management decisions and post-epidemic recovery policies.

Notably, the effective application of big data technology, supplemented by traditional COVID-19 epidemic emergency management measures, requires strong execution and coordination mechanisms within a government and society [37]. Public participation and public compliance are also key drivers of the effectiveness of this application [37, 38]. The authorities need to take extensive publicity measures to increase public understanding, support and adoption before the large-scale promotion of big data interventions for epidemics. Therefore, health intervention measures based on big data technology, such as contact tracing and isolation, need to be limited to a necessary scope, and the public’s right to privacy and informed consent and human rights in general should be fully protected [28, 39].

Specifically, in the process of the COVID-19 epidemic emergency management, the Chinese government has made full use of big data technology in the field of emergency publicity and mobilization to improve public participation and public compliance. The main measures include but were not limited to: 1) applying big data technology to analyse public preferences and to produce and disseminate COVID-19 epidemic emergency management policies and knowledge to improve public health literacy and compliance; 2) applying big data technology to collect general public opinion on both traditional and new media, such as television shows, social media, and short video platforms, to identify public sentiment, adjusting emergency management policies accordingly; and 3) applying big data technology to identify public needs by analysing public opinion and public sentiment, and the authorities accordingly respond to these needs accurately and in a timely manner. As a result of these efforts, the Chinese public has generally formed a consistent understanding of the COVID-19 epidemic emergency management and has a high degree of recognition of the authorities’ decision-making, thereby greatly improving public compliance. In turn, these efforts have promoted the widespread application and public adoption of big data technology in COVID-19 epidemic emergency management in China.

In short, although the benefits of big data technology for the COVID-19 epidemic emergency management have been widely accepted, the process of the technology application needs to improve on its information disclosure, public privacy protection, social compliance, positive role for a government, transparency and accountability while eliminating digital discrimination and any digital divide to augment public trust, adoption, and participation [37–40].

### Prospects of application and practice

This research started by solving the difficulties of emergency management of epidemics in different countries and regions and the limitations of traditional emergency management measures, and proposed an application framework for big data technology to support the COVID-19 epidemic emergency management. The case of Hainan Province further proved that the application framework has high responsiveness and agility and is feasible, efficient and valuable. At the same time, the application framework was adopted to identify high-risk populations, locations and areas to create faster and more accurate strategies. These advantages and conveniences have expanded the scope and scenarios of the framework.

European countries, the United States and other Western countries have struggled to adopt strict epidemic emergency management strategies due to their own unique political, cultural and civic concepts [41–45]. In addition, underdeveloped countries and regions, represented by Africa, may not be able to implement strict emergency management measures in the long term because of their lack of leadership, funds, human resources and medical resources [46, 47]. As a package solution, the application framework proposed in this article hopefully helps these countries and regions resolve these dilemmas due to its agility, scalability and efficiency.

In particular, for island countries and regions where COVID-19 cases are mostly imported, the focus of emergency management measures should be to identify and isolate imported cases and their contacts quickly. The solutions from Hainan Province provide these countries and regions with detailed operating instructions.

For countries and regions that have not yet adopted big data technology or only use it in individual instances and scenarios for their COVID-19 epidemic emergency management, the application framework we proposed can provide them with comprehensive and systematic guidance in their mitigation, preparedness, response and recovery stages. In short, our research results have broad application prospects for different countries and regions and for the whole process of COVID-19 epidemic emergency management.

### Limitations

Our research is limited in the following three aspects: First, the integration of big data technology by local governments into their epidemic emergency management is a gradual process, but our research did not focus on continuous digital transformation. Second, this research divided epidemic emergency management into four stages, i.e., mitigation, preparedness, response, and recovery, and studied the role of big data technology in them, but the basic activities of each stage need to be adjusted according to the characteristics of each particular epidemic and policy environment. Scenario-based applications need to be further improved. Third, the social compliance and security issues of applying big data technology in epidemic emergency management need to be further studied and improved; issues such as proper use, information leakage, privacy protection, transparency and accountability.

## Conclusions

Based on the perspective of emergency management lifecycle theory, this study compared the differences in the process and the effects of epidemic emergency management with and without the support of big data technology and proposed a COVID-19 epidemic emergency management application framework of big data technology with broad application prospects. The aim of this work is to overcome the limitations of the governance heterogeneity that stems from diversity in different countries and regions and traditional epidemic emergency management. Based on the application framework, our research took Hainan Province as a case study to introduce how the Hainan provincial government used big data technology in the four stages of its COVID-19 emergency management: mitigation, preparedness, response and recovery. We then analysed the roles, governance networks and mechanisms used by the legislative branch, government agencies and social organizations of Hainan Province and verified the feasibility and value of the application framework of big data technology to support their collective response to the COVID-19 epidemic.

The application of big data technology by Hainan Province entailed a graph database association graph analysis and the use of the ECharts data visualization tool to track infected populations, suspected infected populations and close contacts to identify high-risk areas and locations. This practice provides a theoretical basis and agile evidence-based support for local governments that decide to implement accurate COVID-19 epidemic emergency management strategies, thereby improving the responsiveness of their COVID-19 epidemic emergency response systems.

The theoretical framework, application framework, mechanism and technology involved in our research could be applicable to various countries and regions in various stages of epidemics emergency management. Clearly identifying and implementing the steps in the continuous process of digital transformations of epidemic emergency management, expanding application scenarios, and resolving social compliance and security issues are the keys to optimizing the application of big data technology for epidemic emergency management.

## Data Availability

The datasets used and/or analysed during the current research are available from the corresponding author upon reasonable request.

## Abbreviations

COVID-19: Corona Virus Disease-19
CDC: Centre for Disease Control and Prevention
